# Supplementary material for: A Promising Intracellular Protein-Degradation Strategy: TRIMbody-Away Technique Based on Nanobody Fragment
Source: Biomolecules. 2021 Oct 14;11(10):1512. doi: 10.3390/biom11101512 (PMC8533776; doi:10.3390/biom11101512)
Supplement: Supplementary file 1 [file biomolecules-11-01512-s001.zip › biomolecules-1396561-supplementary.pdf]

biomolecules

MDPI

Article

Supplementary Files

A Promising Intracellular Protein-degradation Strategy: TRIMbody-Away Technique Based on Nanobody Fragment

Gang Chen [1], Yu Kong [1], You Li [1], Ailing Huang [1], Chunyu Wang [1], Shanshan Zhou [1], Zhenlin Yang [2], Yanling Wu [1,*], Jianke Ren [3,*] and Tianlei Ying [1,*]

[1] MOE/NHC/CAMS Key Laboratory of Medical Molecular Virology, School of Basic Medical Sciences, Shanghai Medical College, Fudan University, 200032 Shanghai, China; 16111010053@fudan.edu.cn (G.C.); kongyu@fudan.edu.cn (Y.K.); zhonghualiyou@163.com (Y.L.); huang_ailing@fudan.edu.cn (A.H.); 15211010049@fudan.edu.cn (C.W.); shanshanzhou1110@163.com (S.Z.)

[2] Department of Pulmonary Medicine, Zhongshan Hospital, Fudan University, 200032 Shanghai, China; yang_zhenlin@fudan.edu.cn (Z.Y.)

[3] CAS Key Laboratory of Computational Biology, Shanghai Institute of Nutrition and Health, University of Chinese Academy of Sciences, Chinese Academy of Sciences, 200031 Shanghai, China

* Correspondence: yanlingwu@fudan.edu.cn (Y.W.); renjianke@picb.ac.cn (J.R.); tlying@fudan.edu.cn (T.Y.); Tel.: +86-021-54237761 (Y.W); +86-021-54920668 (J.R.); +86-021-54237761 (T.Y.)

Citation: Chen, G.; Kong, Y.; Li, Y.; Huang, A.; Wang, C.; Zhou, S.; Yang, Z.; Wu, Y.; Ren, J.; Ying, T. A Promising Intracellular Protein-degradation Strategy: TRIMbody-Away Technique Based on Nanobody Fragment. *Biomolecules* **2021**, *11*, 1512. https://doi.org/10.3390/biom11101512

Academic Editor: Walid A. Houry

Received: 10 September 2021
Accepted: 7 October 2021
Published: 14 October 2021

Publisher's Note: MDPI stays neutral with regard to jurisdictional claims in published maps and institutional affiliations.

Copyright: © 2021 by the author. Licensee MDPI, Basel, Switzerland. This article is an open access article distributed under the terms and conditions of the Creative Commons Attribution (CC BY) license (https://creativecommons.org/licenses/by/4.0/).

Contents:

Supplementary Figure S1

Supplementary Video S1

Supplementary Video S2

Supplementary Figure S2

Supplementary Table S1

Supplementary Figure S3

Supplementary Figure S1

Determination of the optimal Dox concentration.

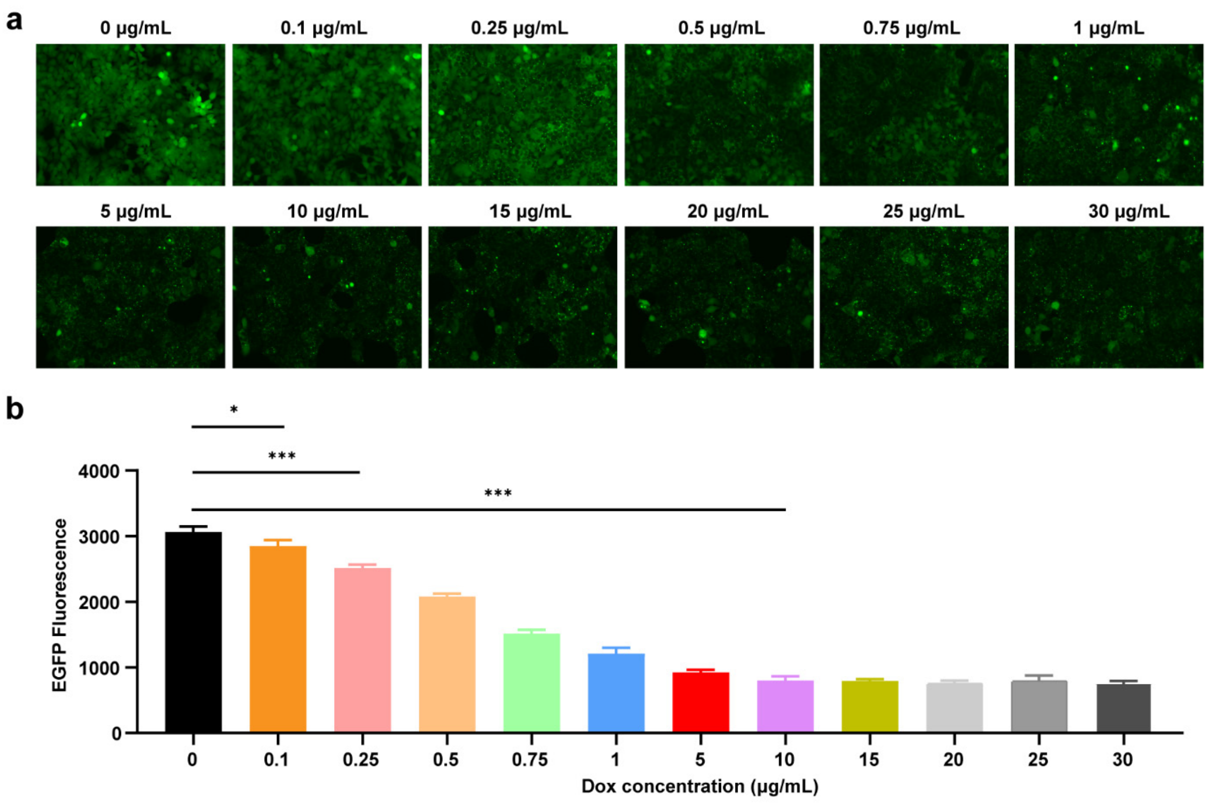

Figure S1. Determination of the optimal Dox concentration. (**a**) Fluorescence microscopy images showing the change in EGFP expression at 48 h in cells untreated or treated with Dox under different concentrations. (**b**) Average fluorescence intensity of EGFP in cells untreated or treated with Dox under different concentrations. Average fluorescence intensity is measured using a flow cytometry and indicated by bar graphs (n = 3 replicates per group). Data represent the mean ± SEM. ns represents no significance, * $P < 0.05$ and *** $P < 0.001$ represents statistical significance.

Supplementary Video S1

Time-lapse movie of dynamic exchange of cytoplasmic EGFP protein, related to Figure 2 c.

Video S1. Time-lapse movie of dynamic exchange of cytoplasmic EGFP protein, related to Figure 2 c. 293T-EGFP/Tet-On-3G-αEGFP TRIMbody cells were treated without Dox for 72 h. Time shows h:min:sec.ms from vehicle or Dox addition. Images obtained with the DeltaVision Elite high-resolution cell imaging systems over a period of ~7 h at 1 frame per 5 or 8 minutes. After imaging is completed, images were processed with softWoRx® Explorer 1.3 software, Adobe Illustrator software and ImageJ software. The cells in the border shows the process of cytoplasmic EGFP protein aggregate into EGFP fluorescent puncta after meiosis. Scale bar, 10 μm.

Supplementary Video S2

Time-lapse movie of dynamic exchange of EGFP protein aggregates between the fluorescent puncta and cytoplasmic pool, related to Figure 3 a.

Video S2. Time-lapse movie of dynamic exchange of EGFP protein aggregates between the fluorescent puncta and cytoplasmic pool, related to Figure 3 a. 293T-EGFP/Tet-On-3G-αEGFP TRIMbody cells were treated with Dox for 24 h. Time shows h:min:sec.ms from vehicle or Dox addition. Images obtained with the DeltaVision Elite high-resolution cell imaging systems over a period of 7 h at 1 frame per 10 minutes. After imaging is completed, images were processed with softWoRx® Explorer 1.3 software, Adobe Illustrator software and ImageJ software. The cells in the border shows a decrease in fluorescence intensity of cytoplasmic EGFP protein after meiosis during Dox induction. Scale bar, 10 μm.

Supplementary Figure S2

The relative mRNA levels of genes related to ubiquitin and autophagy in cells that were untreated or treated with Dox.

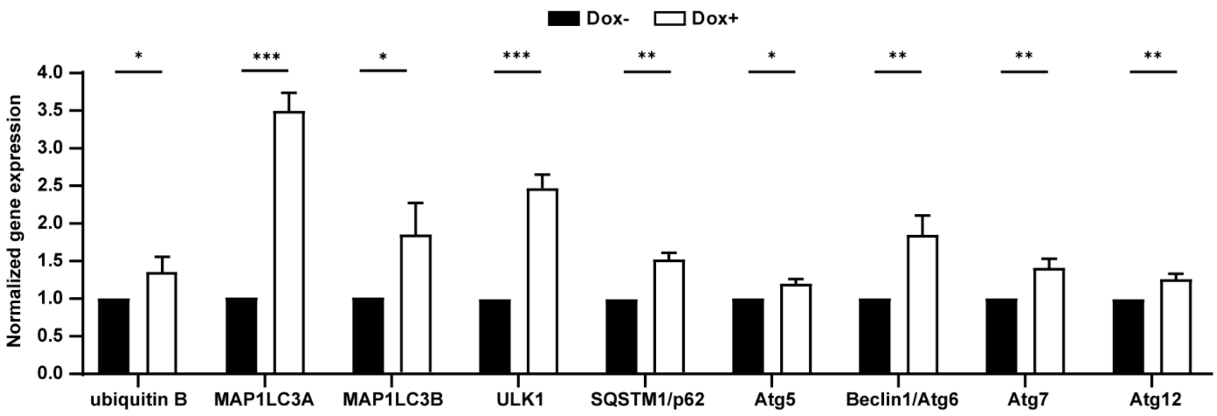

Figure S2. The relative mRNA levels of genes related to ubiquitin and autophagy in cells that were untreated or treated with Dox. Gene expression was normalized to β-actin in each group. * $P < 0.05$, ** $P < 0.01$ and *** $P < 0.001$ represents statistical significance.

Supplementary Table S1

Primers used in this study.

Table S1. Primers used in this study.

Sr.no	Gene ID	Primer	PrimerBank ID	Sequence	Length	Amplicon Size
1	7314	ubiquitin B	22538474c1	F: 5′-GGTCCTGCGTCTGAGAGGT-3′	19	106
				R: 5′-GGCCTTCACATTTTCGATGGT-3′	21	
2	84557	MAP1LC3A	377652329c1	F: 5′-AACATGAGCGAGTTGGTCAAG-3′	21	127
				R: 5′-GCTCGTAGATGTCCGCGAT-3′	19	
3	81631	MAP1LC3B	12383056a1	F: 5′-GATGTCCGACTTATTCGAGAGC-3′	22	167
				R: 5′-TTGAGCTGTAAGCGCCTTCTA-3′	21	
4	8408	ULK1	225637564c1	F: 5′-GGCAAGTTCGAGTTCTCCCG-3′	20	97
				R: 5′-CGACCTCCAAATCGTGCTTCT-3′	21	
5	8878	SQSTM1/p62	214830450c1	F: 5′-GCACCCCAATGTGATCTGC-3′	19	92
				R: 5′-CGCTACACAAGTCGTAGTCTGG-3′	22	
6	9474	ATG5	92859692c1	F: 5′-AAAGATGTGCTTCGAGATGTGT-3′	22	144
				R: 5′-CACTTTGTCAGTTACCAACGTCA-3′	23	
7	8678	Beclin 1/ATG6	187608304c3	F: 5′-ACCTCAGCCGAAGACTGAAG-3′	20	165
				R: 5′-AACAGCGTTTGTAGTTCTGACA-3′	22	
8	10533	ATG7	222144228c2	F: 5′-CTGCCAGCTCGCTTAACATTG-3′	21	216
				R: 5′-CTTGTTGAGGAGTACAGGGTTTT-3′	23	
9	9140	ATG12	290560745c2	F: 5′-TAGAGCGAACACGAACCATCC-3′	21	153
				R: 5′-CACTGCCAAAACACTCATAGAGA-3′	23	

Supplementary Figure S3

Degradation of EGFP by αEGFP TRIMbody in 293T-EGFP-Low/Tet-On-3G-αEGFP TRIMbody cells.

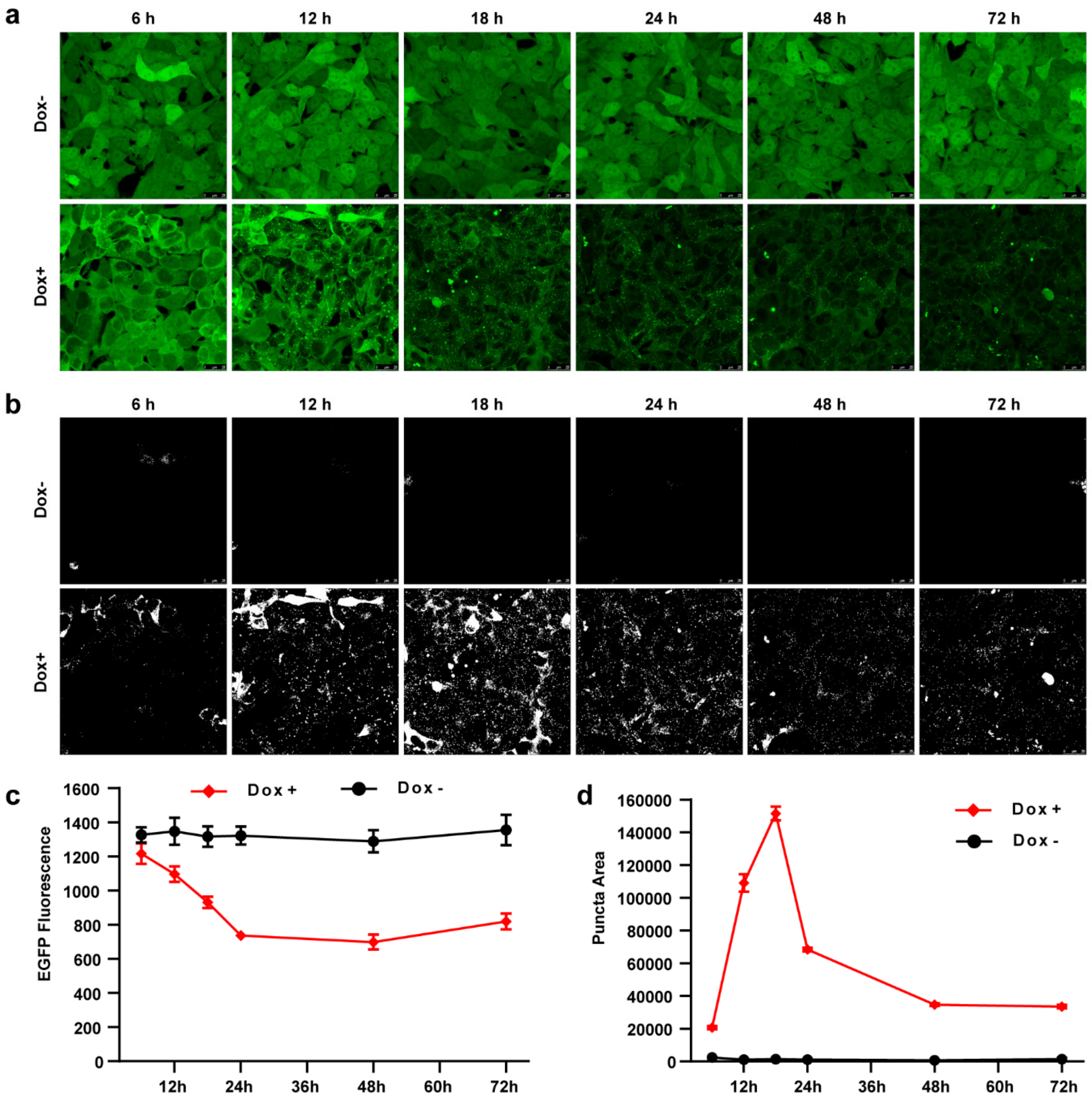

Figure S3. Degradation of EGFP by αEGFP TRIMbody in 293T-EGFP-Low/Tet-On-3G-αEGFP TRIMbody cells. (**a**) αEGFP TRIMbody caused EGFP degradation. Laser scanning confocal fluorescence microscopy images showed the change in EGFP expression over time with and without doxycycline. Scale bars, 25 µm. (**b**) EGFP fluorescent puncta was examined from fluorescent images using Image J software. (**c**) Mean fluorescence intensity of EGFP in cells untreated or treated with Dox. Mean fluorescence intensity was measured using a flow cytometry and indicated by bar graphs (n = 3 replicates per group). Data represent the mean ± SEM. (**d**) Relative EGFP puncta area of autophagosomes or autolysosomes were measured using Image J software. Statistical analysis of the puncta area of autophagosomes and autolysosomes per cell were samples from a pool of at least 3 images. Data represent the mean ± SEM.
